# The 675 nm laser for the treatment of facial acne scars in dark skin type

**DOI:** 10.1111/srt.13514

**Published:** 2023-10-26

**Authors:** Ido Alter, Beatrice Marina Pennati, Francesca Madeddu, Tiziano Zingoni

**Affiliations:** ^1^ Private Clinic Tel Aviv Israel; ^2^ El.En. Group Firenze Italy

To the Editor,

One of the most upsetting and pervasive consequences of acne vulgaris is the development of scars, that can either be proliferative or atrophic. These can have a negative impact on a person's mental, physical, and social well‐being.^1^ Specifically, atrophic acne scars are much more challenging to medicate than other traumatic scar types. Depending on the clinical presentation, a variety of treatment options, including surgical and nonsurgical methods, are available.^2^ Moreover, Light‐emitting diode (LED)‐based devices have recently emerged as one of the most secure and effective ways to treat different skin problems, including skin inflammatory disorders, acne scarring, ageing, and abnormal hair growth. Nevertheless, antibiotics, topical retinoids, azelaic acid, benzoyl peroxide, and isotretinoin^3^ are still the main forms of treatment, although they frequently lead to low compliance, a lack of long‐term remission, and adverse effects. For this reason, lasers are increasingly being used to facial treat acne since they have few side effects, require fewer office‐based treatments, may be beneficial for treating acne scarring at the same time, and produce results quickly. The most common techniques to treat acne and related scarring are the near‐infrared diode lasers, 1450 nm diode laser, the 1550 nm Er:Glass fractional laser and ablative laser therapy (using CO_2_ and Er:YAG lasers), the 585‐ and 595‐nm pulsed dye lasers (PDLs), 1320 nm Nd:YAG laser, 532 nm potassium titanyl phosphate laser, 1064 nm long‐pulsed Nd:YAG laser, 1540 nm Erbium (Er):Glass Laser.^4^ Specifically, the 675 nm wavelength showed successful results in many aspects of facial ageing^5^, ^6^, ^7^ as well as in melasma management,^8^ in dark phototypes^7^, ^9^ and in clinically difficult‐to‐treat populations such as Asians.^10^

Nevertheless, there is not much scientific evidence in the literature reporting treatment for acne scars with the overmentioned wavelength laser in patients with dark phototypes.

With all these premises, we report some exemplificative cases treated to evaluate the efficacy and post‐treatment outcomes of a laser system emitting a 675 nm wavelength in the management of face atrophic acne scars in patients with darker phototypes.

This laser device concentrates its activity on the tissue only in specific areas. Due to the significant depth of action and significant thermal stimulation on the connective component, it is possible to treat the fibrotic tissue that typically affects skin with acne and related scars.

The study aimed to determine the clinical effectiveness of a laser with a wavelength of 675 nm (RedTouch laser, DEKA M.E.L.A., Calenzano, Italy) for the non‐invasive treatment of facial acne scars. For this research, three patients were treated. They were two males and one female patient aged 25 to 59 (mean 42 ± 17). The female patient presented a Fitzpatrick skin type IV while the males were skin type II and IV, respectively. The device uses a 13·13 mm scanning handpiece to emit red light with a wavelength of 675 nm. The treatment settings, reported in Table 1, were power 5–10 W, Dwell time of 200–250 ms, Spacing 1000 μm and Energy/DOT of 1–2.5 J. A total of 5 treatments at 1‐month intervals were performed for every patient. Parameters were adapted to skin type with a decrease the power and, so, the energy/DOT. Moreover, the device is equipped with a contact sensor assuring a safe and optimal treatment. A total of five treatments at 1‐month intervals were performed for every patient. After laser session, water‐soaked gauzes to cool down the skin, and a soothing moisturizing skin emulsion was applied for 2 h after the treatment. Side effects such as blisters, scars, burns, hyperpigmentation and hypopigmentation were monitored during and after the treatment.

**TABLE 1:** Settings used during the treatment with the 675 nm laser.

Technical specifications	
Wavelength	675 nm
Power	5‐10 W
Scan area	13 · 13 mm
Scanning shapes	Square and ellipse
Scan modes	SmartTrack
Dwell time	200–250 ms
Spacing	1000 μm
SmartStack	1
Integrated skin cooler	5°C

Standardized photos were obtained at baseline and at 3 months of follow‐up. The efficacy evaluation was carried out by using a 5‐point scale: no change = 0 point; 25%, 25% mild improvement = 1 point; 50% moderate improvement = 2 points; 75% good improvement = 3 points and 100% excellent improvement = 4 points. The improvement of acne scars was evaluated as a general better appearance of the skin considering the change in smoothing, softening, “glow effect”, and texture.

A Global Aesthetic Improvement Scale (GAIS) with a 5‐point scale (None‐0, Mild‐1, Moderate‐2, Good‐3, Excellent‐4) was used to assess treatment efficacy. Thirty‐three percent of patients have reported good improvements while the majority (67%) reported an “excellent‐4” improvement after the treatment (see Figure 1).

**FIGURE 1:**
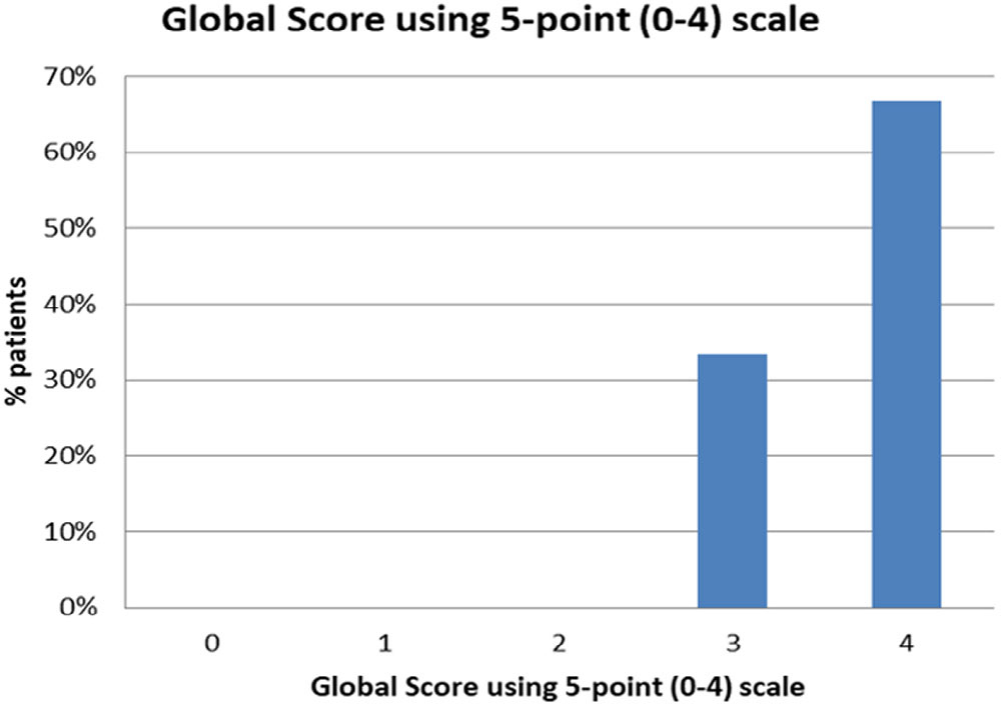
Graphical representation of the GAIS (None‐0, Mild‐1, Moderate‐2, Good‐3, Excellent‐4) for the assessment of patient satisfaction before and after 3 months from the last treatment with the 675 nm laser. GAIS, Global Aesthetic Improvement Scale.

Clinical results are shown in Figures 2 and 3. After 3 months after the last treatment, substantial improvements in the skin texture were visible. The skin appears rejuvenated, and a softening of acne scars has been noticed.

**FIGURE 2:**
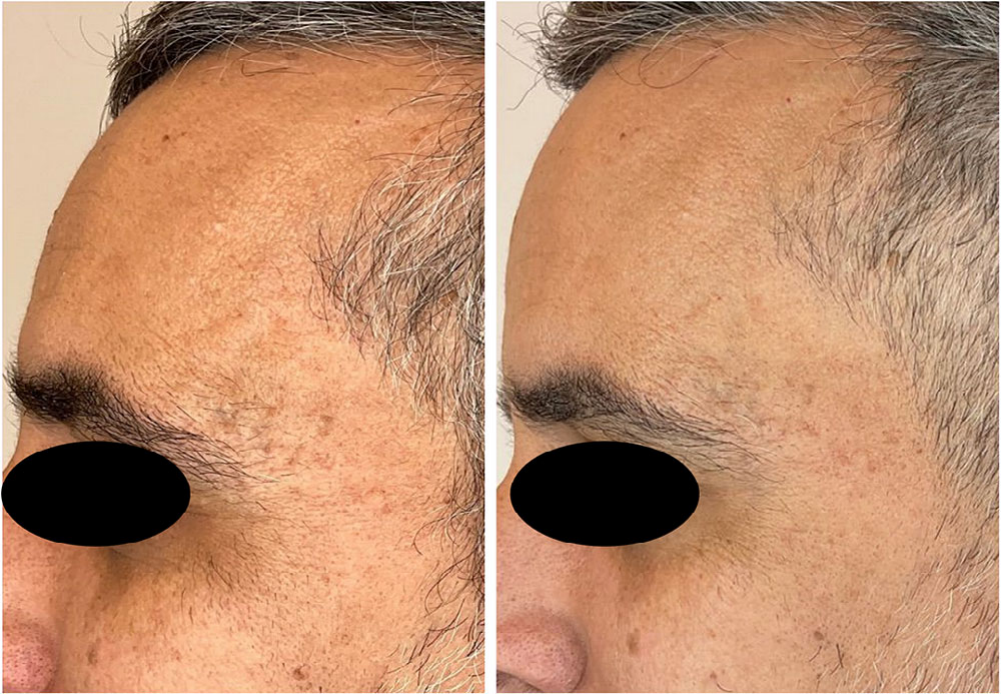
Treatment with the 675 nm laser performed on a male patient, periocular area. The baseline and the follow‐up after 3 months from the last treatment are shown.

**FIGURE 3:**
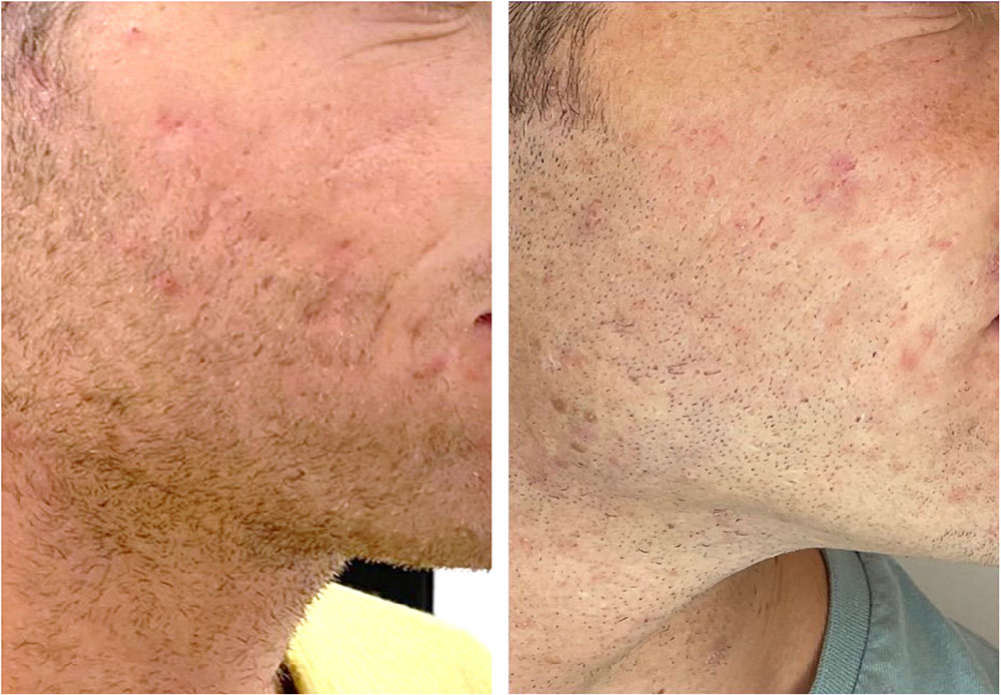
Treatment with the 675 nm laser performed on a male patient, cheek area. The baseline and the follow‐up after 3 months from the last treatment are shown.

The treatment of acne scars remains a challenge aesthetic medicine. Even if many strategies are present to improve skin's appearance, it is important to find other easy‐to‐tolerate options to reduce downtime and adverse effects. When compared to non‐ablative lasers, ablative lasers are more aggressive since they vaporize tissue. On the contrary, when compared to ablative lasers, the possible harmful concerns connected with non‐ablative lasers are significantly lower. Indeed, patients may only show a brief erythema for a few hours without experiencing other adverse effects such as skin peeling or scaling. Since these devices do not cause hyperpigmentation, unlike ablative lasers, they can also be utilized on patients with darker phototypes. The technique used in this study enables many potential pairings of the operating parameters to address acne scarring symptoms. Its wavelength has a strong affinity for collagen fibres and avoids contact with the vascular component of the dermis, making it, in our opinion, a potential therapeutic option for acne scarring. The device additionally includes a skin contact cooling handpiece (5°C) to protect the epidermis from harm brought on by the temperature increase. As a result, the post‐treatment maintenance and the risk of side effects are reduced. As a result, a thermal column forms, which conveys the heat to the nearby areas and causes rapid collagen shrinkage and denaturation as well as the formation of new collagen and consequent scar ‘‘softening’’.

In conclusion, compared to conventional ablative laser treatments, the use of a 675 nm laser device for acne scars guarantees reduced aggression during the treatment. The treated skin shows a general improvement in smoothing, softening, “glow effect” and texture and the recover times are short or zero.

## CONFLICTS OF INTEREST STATEMENT

B.M.P., F.M. and T.Z. are employed at El. En. Group. The authors declare that the research was conducted in the absence of any commercial or financial relationships that could be construed as a potential conflict of interest.

## INSTITUTIONAL REVIEW BOARD STATEMENT

All the authors declare that the procedures followed were in accordance with the Declaration of Helsinki.

## INFORMED CONSENT STATEMENT

Informed consent was obtained from all subjects involved in the study.

## Data Availability

Data is available on request due to privacy restrictions. The data presented in this study are available on request from the corresponding author.
